# Simple and Sensitive Method for the Quantitative Determination of Lipid Hydroperoxides by Liquid Chromatography/Mass Spectrometry

**DOI:** 10.3390/antiox11020229

**Published:** 2022-01-25

**Authors:** Chongsheng Liang, Siddabasave Gowda B. Gowda, Divyavani Gowda, Toshihiro Sakurai, Iku Sazaki, Hitoshi Chiba, Shu-Ping Hui

**Affiliations:** 1 Graduate School of Health Sciences, Hokkaido University, Kita-12, Nishi-5, Kita-Ku, Sapporo 060-0812, Japan; chongsheng.liang.r2@elms.hokudai.ac.jp (C.L.); zaki-ry-0925@eis.hokudai.ac.jp (I.S.); 2 Faculty of Health Sciences, Hokkaido University, Kita-12, Nishi-5, Kita-Ku, Sapporo 060-0812, Japan; siddabasavegowda.bommegowda@hs.hokudai.ac.jp (S.G.B.G.); divyavani@hs.hokudai.ac.jp (D.G.); sakura@hs.hokudai.ac.jp (T.S.); 3 Graduate School of Global Food Resources, Hokkaido University, Kita-9, Nishi-9, Kita-Ku, Sapporo 060-0809, Japan; 4 Department of Nutrition, Sapporo University of Health Sciences, Nakanuma, Nishi-4-3-1-15, Higashi-Ku, Sapporo 007-0894, Japan; chiba-h@sapporo-hokeniryou-u.ac.jp

**Keywords:** lipid hydroperoxide, unsaturated fatty acids, 2-methoxypropene, chemical derivatization, liquid chromatography, mass spectrometry, human serum, lipoprotein oxidation

## Abstract

Lipid hydroperoxides (LOOH) are the initial products of the peroxidation of unsaturated lipids and play a crucial role in lipid oxidation due to their ability to decompose into free radicals and cause adverse effects on human health. Thus, LOOHs are commonly considered biomarkers of oxidative stress-associated pathological conditions. Despite their importance, the sensitive and selective analytical method for determination is limited, due to their low abundance, poor stability, and low ionizing efficiency. To overcome these limitations, in this study, we chemically synthesized eight fatty acid hydroperoxides (FAOOH), including FA 18:1-OOH, FA 18:2-OOH, FA 18:3-OOH, FA 20:4-OOH, FA 20:5-OOH, FA 22:1-OOH, FA 22:6-OOH as analytes, and FA 19:1-OOH as internal standard. Then, they were chemically labeled with 2-methoxypropene (2-MxP) to obtain FAOOMxP by one-step derivatization (for 10 min). A selected reaction monitoring assisted targeted analytical method was developed using liquid chromatography/tandem mass spectrometry (LC-MS/MS). The MxP-labelling improved the stability and enhanced the ionization efficiency in positive mode. Application of reverse-phase chromatography allowed coelution of analytes and internal standards with a short analysis time of 6 min. The limit of detection and quantification for FAOOH ranged from 0.1–1 pmol/µL and 1–2.5 pmol/µL, respectively. The method was applied to profile total FAOOHs in chemically oxidized human serum samples (*n* = 5) and their fractions of low and high-density lipoproteins (*n* = 4). The linoleic acid hydroperoxide (FA 18:2-OOH) and oleic acid hydroperoxide (FA 18:1-OOH) were the most abundant FAOOHs in human serum and lipoproteins. Overall, our validated LC-MS/MS methodology features enhanced detection and rapid separation that enables facile quantitation of multiple FAOOHs, therefore providing a valuable tool for determining the level of lipid peroxidation with potential diagnostic applications.

## 1. Introduction

Lipid peroxidation has attracted much attention in determining the nutritional value of foods and potentially influences pathophysiological processes, including sarcopenia, aging, and inflammatory diseases [1,2,3]. Lipid hydroperoxides (LOOHs) are the primary products of unsaturated lipid oxidation, with -OOH moiety is generally being allylic to double bond. Both clinical and animal studies have shown a positive association between LOOHs and disease pathology [4,5]. Moreover, long-term fish oil consumption creates a high risk for membrane phospholipid peroxidation and produces LOOHs, increasing senescence [6]. Since lipids play a crucial role in food lipid quality management, their oxidation affects the shelf-life of food. The formation of lipid oxidation products in food depends on both internal (ex: fatty acid composition) and external factors (ex: temperature, light, moisture level). Further, lipid oxidation products are responsible for off-flavor foods and cause diseases, such as inflammation, cancer, atherosclerosis, and aging in humans [7,8,9]. Therefore, the quantity and the actions of LOOHs in vivo should be known to evaluate exactly their biological effect. Hence, accurate determination of LOOHs is essential for finding and thereby control of the oxidation process in disease conditions. Concentrations of LOOHs in human serum and lipoproteins have been ambiguous because of the difficulties in their measurements. Previous studies were focusing on the determination of phospholipid, glycerolipid, and cholesterol hydroperoxides with the aid of high-performance liquid chromatography (HPLC) with UV detection or with the state of the art of mass spectrometry [10,11,12]. In addition to this, electron paramagnetic resonance spin-trapping and nuclear magnetic resonance techniques are also applied for measurements of hydroperoxides [13,14]. Nevertheless, these techniques are limited by their sensitivity, requirement of a high amount of sample, and significant interference of unoxidized lipids.

Furthermore, the chemical stability of LOOHs is a factor of concern. Because LOOHs are the primary oxidation products that are easily transformed into secondary products in an open environment. Despite the authentic standards stored at −30 °C the stability of LOOHs was not last more than 3 months [15,16] Moreover, often biological samples were analyzed in large numbers to obtain statistically significant results, and this increased the sample waiting time in autosampler, which could cause the degradation of LOOHs and affect the accuracy of the experiment results. Liquid-chromatography/mass spectrometry (LC/MS) is a robust technology widely applied for lipidomic analysis because of its high sensitivity over conventional techniques. The past studies demonstrated the application of the LC/MS technique to LOOHs analysis [17,18,19]. They are limited by the poor ionization efficiency of LOOHs, lack of detailed mass fragmentation specific to -OOH moiety, and require large sample volume. An earlier study demonstrated the stability enhancement and analysis of LOOHs by derivatizing with 2-methoxypropene [16]. However, the ionization efficacy of 2-MxP derivatives and their mass behaviors were not evaluated in detail. In this study, we have chemically synthesized eight fatty acid hydroperoxides (FAOOHs) and their 2-MxP derivatives (FA-OOMxP) to develop a facile LC/MS method for quantitative analysis. FA-OOMxP is comparatively more stable than FAOOHs and undergoes a strong ionization in positive mode to give unsaturation specific mass fragment ions. The targeted single reaction monitoring channels for each molecular species were established using a triple quadrupole mass spectrometer and robust analytical method. After validating the method, it was successfully applied to profile the total FAOOHs in oxidized human serum and lipoproteins.

## 2. Materials and Experimental

Linoleic acid, pyridine, hematoporphyrin, dichloromethane, chloroform, and pyridinium p-toluene sulfonate, were purchased from Wako Pure Chemical Industry, Ltd., (Tokyo, Japan). Oleic acid, γ-linolenic acid, arachidonic acid, all cis-5,8,11,14,17-eicosapentaenoic acid, cis-4,7,10,13,16,19-docosahexaenoic acid, 2-methoxypropene were purchased from Tokyo Chemical Industry Co., Ltd., (Tokyo, Japan). cis-10-Nonadecenoic acid and human plasma (Cat. No. P9523) were purchased from Sigma Aldrich (St. Louis, MO, USA). Erucic acid was purchased from Alfa Aesar (Tewksbury, MA, USA). Hexane, ethyl acetate, acetonitrile, methanol, tert-Butyl Methyl Ether, and all other reagents of solvent grade were obtained from Kanto Chemical Co. Ltd., (Tokyo, Japan).

### 2.1. General Procedure for the Synthesis of FAOOH and FAOOMxP Standards

**Figure 1:**
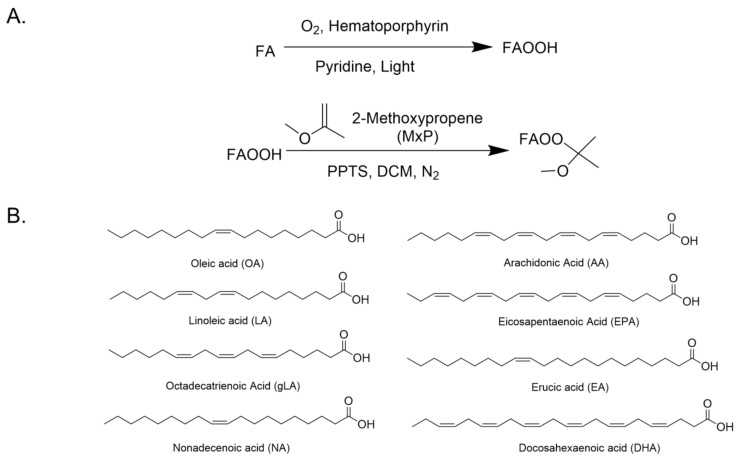
Synthesis of fatty acid hydroperoxides and their 2-MxP derivatives (**A**) Schematic representation of the synthesis of FAOOH and FAOOMxP. (**B**) Chemical structure of unsaturated fatty acids used in the study.

Synthesis of fatty acid hydroperoxides (FAOOH): The synthesis of FAOOHs was achieved by the photochemical oxidation method established earlier with minor modifications [20]. The reaction scheme and structures of fatty acid standards used are summarized in Figure 1. Briefly, the respective unsaturated fatty acid was dissolved in pyridine along with a pinch of hematoporphyrin (~0.005 eq). Then the reaction mixture is irradiated under a 200 W tungsten lamp with continuous bubbling of O_2_ gas for 75–90 min at 15 °C. The progress of the reaction was monitored by thin-layer chromatography (TLC). After completion, the reaction mixture was poured into ice-cold water, acidified with 2 M HCl, and extracted supplwith dichloromethane. The combined organic layer was washed with brine and dried over by anhydrous sodium sulfate. After evaporation, the crude product was subjected to silica gel column chromatography (hexane/ethyl acetate (*v*/*v*) = 48/2 to 35/15) for further purifications. The details on the synthesis of individual FAOOH standards were provided in the supporting information (Supplementary material Pages S1–S3).

Synthesis of 2-Methoxypropene (2-MxP) derivatized fatty acid hydroperoxides (FAOOMxP): The purified FAOOHs were subjected to 2-MxP derivatization by the previously reported protocol with minor modifications [16]. In brief, the respective FAOOH was dissolved in dichloromethane, and a catalytic amount of Pyridinium p-Toluene sulfonate (PPTS) and an excess amount of 2-MxP were added. The reaction was stirred at room temperature for 10 min under a nitrogen atmosphere, for completion. The reaction mixture was poured into water and extracted with dichloromethane. The combined organic extracts were washed with brine solution, dried over anhydrous sodium sulfate, and subjected to column chromatography for purification. The experimental details of all the FAOOMxP standards are provided in supporting information (Supplementary material Pages S3–S5).

### 2.2. Analysis of FAOOMxPs by Targeted LC-MS/MS

The single reaction monitoring (SRM) channels for each FAOOMxP were established using TSQ Quantum Access MAX Triple Quadrupole Mass Spectrometer (Thermo Fisher Scientific, Inc., Waltham, MA, USA) in positive ionization mode. The optimized ion source parameters were set as follows: spray voltage:3500 V, vaporizer temperature: 150 °C, capillary temperature: 250 °C sheath gas (nitrogen): 40 psi, auxiliary gas (nitrogen): 25 psi, and collision gas (argon): 1.5 mTorr respectively. The obtained SRM channels for each FAOOMxP are provided in Table 1. Moreover, the separation was achieved using an ultra-fast liquid chromatograph (UFLC) system (Shimadzu Corp., Kyoto, Japan) equipped with a Hypersil GOLD C4 column (50 mm× 2.1 mm, 1.9 μm, Thermo Fisher Scientific Inc., Waltham, MA, USA). The oven and sample tray temperature were maintained at 40 °C and 4 °C, respectively. The mobile phases were A: Acetonitrile: Milli-Q (1:3) with 0.1% Acetic acid B: Methanol. The gradient at flow rate of 0.3 mL/min was set as follows: 0–1 min (40% A, 60% B), 1–4.5 min (10% A, 90% B), 4.5–5.5 (100% B), 5.5–8 (100% B), and re-equilibration for 2 min.

### 2.3. Ethical Approval

The human serum samples were collected from healthy volunteers (*n* = 5) with prior ethical approval from the ethics committee of the Faculty of Health Science, Hokkaido University. The approval number is 19-107-2. Informed consent was obtained from all subjects involved in the study.

### 2.4. Extraction and Derivatization of Sample

Human serum (50 μL) or lipoproteins (high-density lipoprotein (HDL): 1 mg/mL, low-density lipoprotein (LDL): 0.2 mg/mL), samples were taken in a 1.5 mL Eppendorf and 10 μL of 100 μM internal standard FA19:1-OOH was added. The extraction was performed by adding 200 μL methanol, 800 μL chloroform with vortex for 3 min at 3500 rpm at room temperature by previously established method with modifications [21]. Additionally, 100 μL Milli-Q was added and vortexed for an additional 3 min. Then samples were subjected to centrifugation for 5 min at 15,000 rpm and 4 °C. The organic layer was transferred into a new vial and the aqueous layer was re-extracted with 800 μL tert-Butyl Methyl Ether (TBME). The combined organic extracts are evaporated under a vacuum. The lipid residue was re-dissolved in 100 μL acetonitrile and 50 μL PPTS (1 mM, in ACN), 50 μL 2-MxP were added with vortex for 10 min at room temperature. After that, it spiked 100 μL MilliQ to stop this reaction. The MxP labeled products were extracted with 400 μL of TBME (x2) with vertexing for 3 min and centrifuged for 5 min. The TBME layer was dried in a vacuum, re-dissolved in 50 μL MeOH. The alkali hydrolysis was conducted to determine the total FAOOHs by the modified conditions described earlier [22]. Briefly, to the redissolved 50 μL MeOH extract 10 μL 1 M methanolic KOH was added, vortexed for 3 min, and incubated at 37 °C for 60 min. Then the extracts are cool down and neutralized with 2 μL acetic acid, make up to 100 μL using methanol, and transferred to LC/MS vials. Each injection was set to 10 μL.

### 2.5. Method Validation

To evaluate the linearity of this method, a series of diluted fatty acid hydroperoxides (FA-OOHs) standard solutions were prepared at concentrations 0.01, 0.1, 1, 2.5, 5, 7.5, 10, 25, 50, and 100 pmol/μL with a constant amount of internal standard (FA19:1-OOH, 100 μM) and derivatized with 2-MxP as described in the earlier section. The limit of quantification (LOQ) and limit of detection (LOD) were evaluated as the signal-to-noise (S/N) ratios 10 and 3, respectively. The recovery, matrix effect, and accuracy of the method were evaluated by spiking the known concentration of FAOOH standards (50 pmol/μL) to a 50 μL human plasma and derivatized with 2-MxP. Recovery is calculated by the ratio of the area (spiked sample) to the area (spiked extract) and the matrix effect by area (spiked extract) to the area (standard). The details of these calculations are provided in our earlier report [17,18,19].

### 2.6. Chemical Oxidation of Native Human Serum and Lipoproteins

The oxidation experiments were performed based on the previously reported protocol with minor modifications [11,12]. Briefly, total lipoprotein fraction (density < 1.225) was separated by ultracentrifugation, and then low-density lipoprotein (LDL) and high-density lipoprotein (HDL) were separated using gel-filtration HPLC based on the previously established method with minor modifications [23]. 50 μL of native human serum was mixed with 10 μL of 10 mM copper sulfate and 10 μL of 3.4% hydrogen peroxide. The oxidation was carried out for 24 h at 4 °C. Similarly, to the 150 μL of HDL (1.0 mg/mL) and LDL (0.2 mg/mL), 10 μL of 1 mM copper sulphate and 10 μL of 3.4% hydrogen peroxide were added and oxidized for 2 h at 4 °C.

### 2.7. Statistical Analysis

The data were plotted in Microsoft office Excel 365 and GraphPad Prism 8.0.1. Two-way ANOVA with Tukey or Sidak multiple comparison tests were applied. The significance levels were decided as follows: GraphPad (GP) value of 0.1234 (ns), 0.0332 (*), 0.0021 (**), 0.0002 (***), 0.0001 (****).

## 3. Results

### 3.1. Preparation and Characterization of FAOOH and FAOOMxP Standards

The FAOOH and their 2-MxP derivatives (FAOOMxP) were prepared and purified as described in the experimental section. The authentic standards such as FA 18:1-OOH, FA 18:2-OOH, FA 18:3-OOH, FA 19:1-OOH, FA 22:1-OOH, FA 20:4-OOH, FA 20:5-OOH, and FA 22:6-OOH were obtained from their respective FAs as starting materials by the hematoporphyrin catalyzed photochemical oxidation. The reactants and byproducts of the reaction are removed by silica gel column chromatographic purification. The FAOOHs were further derivatized with excess 2-MxP in the presence of the catalytic amount of PPTS and purified the products (FA 18:1-OOMxP, FA 18:2-OOMxP, FA 18:3-OOMxP, FA 19:1-OOMxP, FA 22:1-OOMxP, FA 20:4-OOMxP, FA 20:5-OOMxP, and FA 22:6-OOMxP) by column chromatography. FAOOH and FAOOMxP were obtained in milligram scale ranging from 5–28 mg and 6–44 mg (of yield about 61%), respectively. Finally, their structures were characterized by nuclear magnetic resonance (NMR) spectroscopy and high-resolution electrospray ionization mass spectrometry (HR-ESI-MS). The linear ion trap quadrupole-Orbitrap mass spectrometry was used to acquire HR-ESI-MS spectra by the conditions mentioned in our earlier report [24].

**Figure 2:**
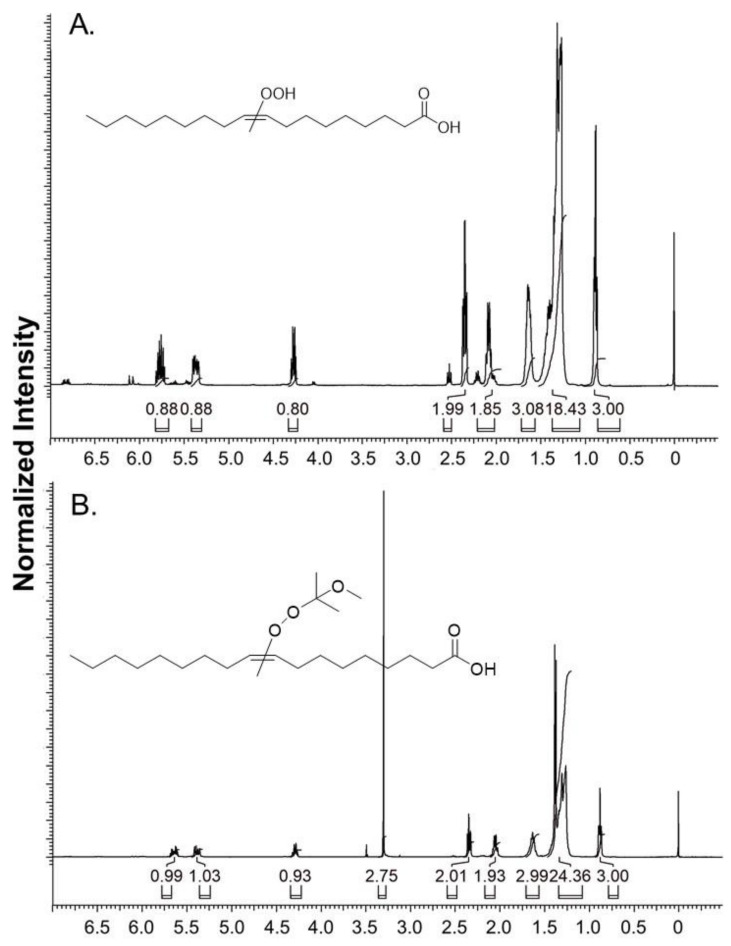
The 1H-NMR spectrum of FA 18:1-OOH (**A**) and its 2-MxP derivative FA 18:1-OOMxP (**B**).

The representative ^1^H NMR spectra for oleic acid-OOH (FA 18:1-OOH) and FA 18:1-OOMxP are provided in Figure 2. The 1H NMR and accurate mass details are as follows: 1H-NMR of FA 18-OOH (400 MHz, CDCl3); δ 5.81–5.72 (m, 1H), 5.41–5.33 (m, 1H), 4.30–4.24 (q, 1H, J = 6.4 Hz, 14.6 Hz), 2.37–2.33 (m, 2H), 2.11–2.06 (m, 2H), 1.66–1.60 (m, 3H), 1.46–1.27 (m, 19H), 0.90–0.86 (m, 3H). The exact mass obtained for FA 18:1-OOH is *m*/*z* 313.2384 (theoretical *m*/*z* calculated for C18H33O4 [M-H]^−^ is 313.2384, mass error: 0 ppm). 1H-NMR of FA18:1-OOMxP (400 MHz, CDCl3) δ 5.69–5.60 (m, 1H), 5.42–5.35 (m, 1H), 4.32–4.26 (q, 1H, J = 6.8 Hz, 14.2 Hz), 3.30 (s, 3H), 2.37–2.32 (m, 2H), 2.08–2.03 (m, 2H), 1.67–1.62 (m, 3H), 1.44–1.26 (m, 25H), 0.90–0.87 (m, 3H). The experimental exact mass obtained for FA 18:1-OOMxP is *m*/*z* 409.2917 (theoretical *m*/*z* calculated for C22H42NaO5 [M + Na]^+^ is 409.2924, mass error: (−1.71 ppm). The formation of -OOH and -OOMxP is confirmed by the NMR peaks at δ 4.3 (-CH-OOH) and δ 3.3 (-OCH3), respectively.

**Figure 3:**
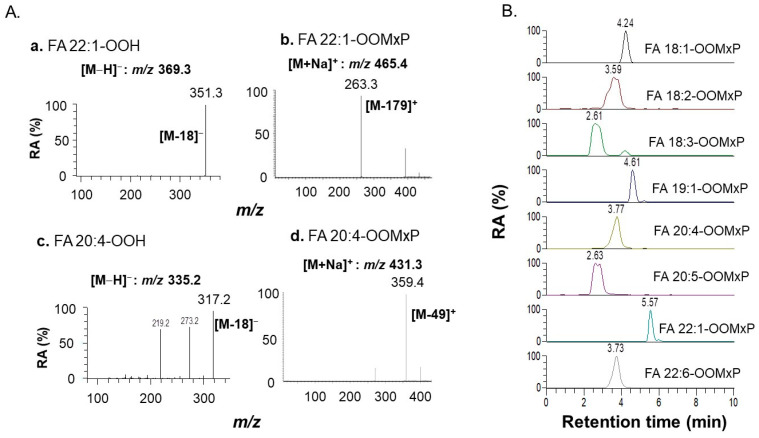
The MS/MS behavior of representative species and elution profile of 2-MxP derivatives. (**A**) The MS/MS spectra of mono-unsaturated (FA 22:1) and poly-unsaturated (FA 20:4) fatty acid hydroperoxides (**a**,**c**) and their 2-MxP derivatives (**b**,**d**). (**B**) Extracted ion chromatograms of FAOOMxP standards (in plasma matrix).

The ionization pattern of mono and poly-unsaturated FAOOMxP derivatives was evaluated. The representative MS/MS spectra of erucic acid hydroperoxide (FA 22:1-OOH), arachidonic acid hydroperoxide (FA 20:4-OOH), and their 2-MxP labeled derivatives (FA 22:1-OOMxP, FA 20:4-OOMxP) are shown in Figure 3A. The previous studies were focused on the identification of LOOHs by the characteristic loss of a neutral molecule of water, nonetheless, this loss is common in lipids other than hydroperoxides like hydroxy fatty acids causing a significant interference in their annotation [18]. In our method, 2-MxP derivatized FAOOHs producing major fragment ions by the characteristic loss of *m/z* 179 and *m/z* 49 (Figure 3A) for mono- and poly-unsaturated FAOOMxP, respectively. These ions are possibly produced by the successive loss or rearrangements of -MxP moiety attached to the -OOH group. The formation of unsaturation-specific fragment ions certainly takes advantage of the conventional method of identification of FAOOHs (by the neutral loss of water). The spectral details of the rest of the available standards and their exact masses are provided in supporting information (Supplementary material Pages S6–S11).

### 3.2. Linearity, Sensitivity, Separation, and Extraction of FAOOH

The results of linearity, the limit of detection (LOD), and the limit of quantification (LOQ) are provided in Table 2. All the standards showed good linearity of R^2^ > 0.97. The LOD and LOQ for FAOOH are range from 0.1–1 pmol/µL and 1–2.5 pmol/µL, respectively. These results suggest sufficient sensitivity for the determination of FAOOHs at the low picomolar level.

The separation was achieved within a period of 6 min using conditions as described in the methods section. The representative extracted ion chromatograms of all the synthesized FAOOMxP are shown in Figure 3B. The high sensitivity and rapid analysis suggest the established method is robust with potential clinical applications. Though FAOOMxP derivatives are successfully prepared from FAOOHs on a large scale, the major challenge with us was the 2-MxP derivatization of FAOOHs at a minor scale as present in biological samples. To achieve this various reaction parameters such as volume of 2-MxP, amount of PPTS, reaction time, and temperature were evaluated by spiking internal standard FA19:1-OOH in plasma matrix. The results are shown in supporting information (Supplementary material page S12). These results demonstrated that the following conditions are ideal for 2-MxP derivatization of FAOOHs in biological samples: 2-MxP (50 μL), 1 mM PPTS (50 μL, in ACN), 10 min at room temperature. The analysis results of the recovery and reproducibility test are listed in Table 3. The coefficients of variation (CV) of intra-day and inter-day assays for all FAOOHs species are <15%. The recoveries of FAOOH after 2-MxP derivatization is range from 48 to 76%. The matrix effect of the internal standard is observed to be 91%, suggesting a slight ion suppression in positive mode. The authentic FAOOH standards (50 pmol/μL) are spiked to plasma and the matrix effect was evaluated. The FA 18:1-OOH and FA 22:1-OOH showed ion enhancement (102% and 120%) whereas all other species showed ion suppression (<100%) with plasma matrix.

### 3.3. Application to Profile FAOOHs in Chemically Oxidized Human Serum and Lipoproteins

**Figure 4:**
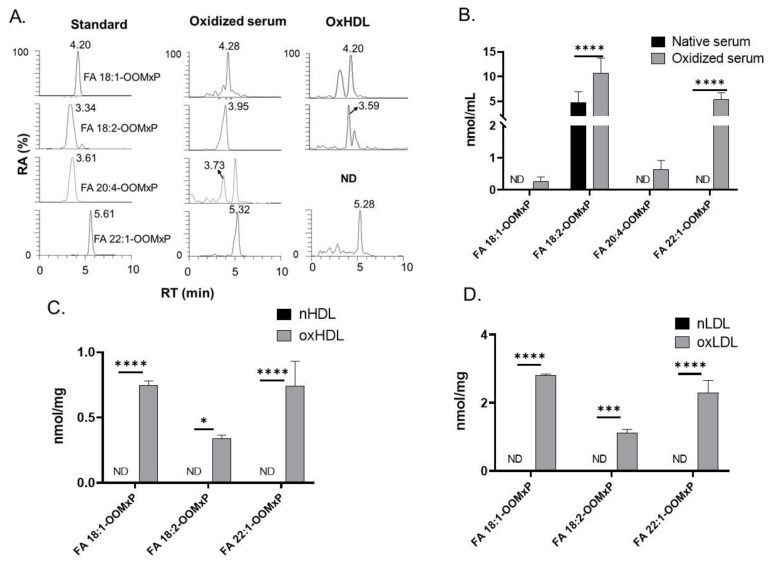
Quantitation of total FAOOHs in human serum and lipoproteins. (**A**) Extracted ion chromatograms of standard and those detected in samples. (**B**) Detected FAOOHs in native and oxidized human serum (*n* = 5). (**C**) Amount of FAOOHs detected in native LDL (nLDL) and oxidized LDL (oxLDL) (*n* = 4). (**D**) Amount of FAOOHs detected in native HDL (nHDL) and oxidized HDL (oxHDL) (*n* = 4). Two-way ANOVA with Tukey multiple comparison tests were applied and *p* < 0.05 was considered to be statistically significant (ND: not detected, *p*= 0.0332 (*), 0.0002 (***), 0.0001 (****)).

After validating the method, it was applied to determine the total FAOOHs in chemically oxidized human serum and lipoprotein (HDL and LDL) samples. The chromatograms of detected species and their quantitative results are shown in Figure 4. In human serum, the detected lipid species were FA 18:1-OOH, FA 18:2-OOH, FA 20:4-OOH, and FA 22:1-OOH, with FA 18:2-OOH being the most predominant. Compared to native serum, the oxidized serum had significantly higher amounts of these oxidized lipids. Further in both HDL and LDL, FA 18:1-OOH, FA 18:2-OOH, and FA 22:1-OOH were the key detected lipid species, with FA 18:1-OOH being most predominant. Compared to native HDL and LDL, the oxidized forms (oxHDL and oxLDL) had significantly higher FAOOHs.

## 4. Discussion

Lipid oxidation by enzymatic and non-enzymatic biological processes generates lipid hydroperoxides (LOOHs), which are major initial products of free-radical-initiated peroxidation of unsaturated fatty acids. LOOHs are reported to play a crucial role in human disease progression [1,2,3]. Thus, their determination in biological samples is of great interest. Several methods of determination of LOOHs are developed, but because of poor stability of -OOH moiety, most are indirect or general methods rather than the analysis that are sensitive and specific for intact LOOHs [25,26,27]. Although the identification of specific LOOHs is achieved by high-performance liquid chromatography coupled to UV detection [28] this method detection is limited to low nanogram levels, and interference of unoxidized lipids causes difficulty in identification. An effort to measure hydroperoxides by gas-chromatography mass spectrometry, derivatization reaction resulted in decomposition of -OOH moiety [29]. Recently, LC/MS analysis has been attracted great concern because of its sensitivity and direct measurements of LOOHs [16,30,31]. Most of these reported techniques are semi-quantitative and not focused on the measurement of the fatty acid hydroperoxides (FAOOHs). To know the significance of FAOOHs, absolute quantitation methods are necessary, however, they are limited due to a lack of authentic standards, instability, and poor mass ionization. To overcome this problem in this study, we synthesized eight authentic standards including, FA19:1-OOH molecular species as an internal standard. Simple photochemical oxidation was employed to prepare FAOOHs followed by 2-MxP derivatization to give FAOOMxP by the method reported earlier with minor modifications [16]. As for our knowledge, this is the first report on the synthesis and characterization of FAOOH and stable FAOOMxP derivatives which are used for quantitative. Previous reports had demonstrated the stability enhancement and separation of LOOHs by 2-MxP derivatization [16,32] The MS/MS analysis confirmed the unsaturation specific fragment ions by the loss of 179 Da and 49 Da for mono- and poly-unsaturated FAOOMxPs, suggesting enhanced detection by positive ionization. The data of LOD ranges from 0.1–1 pmol/µL, and LOQ from 1–2.5 pmol/µL showed that our method is sensitive for FAOOH determination. This sensitivity enables us to detect a small amount of FAOOHs in the human samples. However, the LOD and LOQ are still higher compared to the previous report on the determination of cholesterol ester and phosphatidylcholine hydroperoxides [16,32]. The developed method is still advantageous over the previous method because multiple fatty acyls can be measured with simple extraction and derivatization technique. Furthermore, the detailed method validation was performed, as demonstrated in Table 3, showing that the recoveries are up to 76% with a low coefficient variance, suggesting the high precision of our method. The possible reason for least recoveries of some standards could be a reduction of spiked FAOOHs to FAOH in plasma by the action of endogenous peroxidases [12]. Furthermore, LOOHs are very unstable to alkali hydrolysis as they are reduced to hydroxides [33]. To liberate the FAOOHs from complex lipids, first, the -OOH group was masked with 2-MxP derivatization, and then alkali hydrolysis was conducted. The alkaline stability of FAOOMxPs was evaluated under similar experimental conditions as that of samples and the results were provided in supporting information (Supplementary material Page S13) suggested strong stability of 2-MxP derivatives to alkali. FAOOHs are reported to be increased in oxidatively modified plasma with the increasing course of oxidation [12], and the results are consistent with our study. The chromatograms of samples before and after derivatization demonstrated 2-MxP derivatization enhanced the detection of FAOOHs (Supplementary material Page S14). Compared to native serum or lipoproteins, oxidized serum or oxidized lipoproteins have a higher amount of FAOOHs. A previous study determined the human plasma concentrations of phosphatidylcholines (PC) and found the higher concentrations of PC (16:0/18:2-OOH) (PC 18:0.18:2-OOH) [10]. That could account for the predominance of FA 18:2-OOH species in serum and our analysis results are consistent with this report. The intact LOOHs such as cholesterol hydroperoxide (CE-OOH) and phospholipid (PL-OOH) were characterized in intact as well as oxidized HDL and LDL [30,34,35] However, there are no reports on total FAOOH levels in HDL and LDL. Our results demonstrated the occurrence of a large amount of FA18:1-OOH and FA 22:1-OOH in both oxHDL and oxLDL respectively. The limitation of this study is isomeric species of each standard are not addressed, as it is very difficult to prepare and separate each hydroperoxide regio-isomers [1]. The specific labeled internal standard of each species was not used for absolute quantitation due to lack of commercial availability or difficulty in the preparation process. Hence, the study limits the guidelines of the Food and Drug Administration (FDA) for absolute quantitation of analytes. The derivatization was performed after extraction not before extraction due to solvent compatibility for derivatization which limits this technical application in unextracted samples. Further, the proposed method can determine only fatty acyls having one -OOH group but fatty acyls with oxidation at multiple sites are not uncovered. There is a great possibility of the formation of by-products during the alkali hydrolysis of 2-MxP derivatized total lipids which are not explored in this study. Further, experiments are necessary such as the use of specific enzymatic hydrolysis to eliminate the byproducts of chemical hydrolysis.

## 5. Conclusions

Lipid hydroperoxides may be useful as clinical markers of lipid peroxidation and oxidative stress in the circulation system. Herein, we developed a facile method for determining FAOOHs by 2-MxP derivatization and targeted analysis using state-of-art of mass spectrometry. The simple extraction, rapid separation, and high sensitivity to low picomolar levels suggest our robust technology for FAOOHs measurements. Furthermore, application to the profile of FAOOHs in native human serum, HDL and LDL indicate the predominance of FA 18:2-OOH and FA 18:1-OOH, respectively. In addition, the chemical oxidation of serum and lipoproteins enhanced the FAOOH levels. Hence, the proposed methodology could be a valuable tool for determining lipid peroxidation, evaluating antioxidant potential, and future clinical diagnostic applications.

## Figures and Tables

**Table 1:** Optimized SRM parameters for 2-methoxyprepene derivatized fatty acid hydroperoxides (FA: Fatty acid; MxP: 2-methoxypropene).

Lipids	Parent Ion [M + Na]^+^ (*m*/*z*)	Product Ion (*m*/*z*)	Collision Energy (V)	Tube Lens (V)
FA 18:3-OOMxP	405.3	333.3	11	57
FA 18:2-OOMxP	407.3	335.3	13	57
FA 18:1-OOMxP	409.2	207.3	14	57
FA 19:1-OOMxP (IS)	423.4	221.3	14	62
FA 20:5-OOMxP	429.3	357.3	12	64
FA 20:4-OOMxP	431.4	359.4	13	64
FA 22:6-OOMxP	455.3	383.4	12	53
FA 22:1-OOMxP	465.4	263.3	15	65

**Table 2:** Determination of linearity, LOD, and LOQ of FAOOH standards (^#^ LOD: limit of detection, ^$^ LOQ: limit of quantification).

Lipids	Linearity	R^2^	Range (pmol/µL)	LOD (pmol/µL) ^#^	LOQ (pmol/µL) ^$^
FA 18:3-OOH	0.0003x + 0.0386	0.965	2.5–100	1	2.5
FA 18:2-OOH	0.0022x + 0.0141	0.998	2.5–100	1	2.5
FA 18:1-OOH	0.0026x + 0.2529	0.987	1–100	0.1	1
FA 20:5-OOH	0.0021x + 0.1837	0.979	2.5–100	1	2.5
FA 20:4-OOH	0.0017x + 0.018	0.988	2.5–100	1	2.5
FA 22:6-OOH	0.0008x − 0.003	0.994	1–100	0.1	1
FA 22:1-OOH	0.0022x + 0.0868	0.990	1–100	0.1	1

**Table 3:** Recovery and reproducibility of FAOOH standards in plasma matrix (CV: coefficient of variance) ^#^ The value corresponds to the variance among FAOOH standards derivatized and injected directly without matrix.

Lipids	Recovery (%)	Standard (CV%) ^#^	Intra-Day (CV%)	Inter-Day (CV%)
FA 18:1-OOH	69.7 ± 4.5	2.9	3.1	4.4
FA 18:2-OOH	53.9 ± 4.3	1.2	6.4	8.8
FA 18:3-OOH	54.8 ± 6.0	8.8	9.3	3.9
FA 19:1-OOH	70.9 ± 11.4	8.9	13.6	7.1
FA 20:4-OOH	48.1 ± 5.7	9.7	3.1	5.5
FA 20:5-OOH	50.0 ± 7.5	12.3	7.3	5.2
FA 22:1-OOH	76.0 ± 5.95.	5.1	3.7	3.6
FA 22:6-OOH	49.5 ± 5.6	9.2	4.9	10.9

## Data Availability

The data presented in this study are available in article and supplementary material.

## Supplementary Materials

The following supporting information can be downloaded at: https://www.mdpi.com/article/10.3390/antiox11020229/s1.

## References

[1] Pharaoh G., Brown J.L., Sataranatarajan K., Kneis P., Bian J., Ranjit R., Hadad N., Georgescu C., Rabinovitch P., Ran Q. (2020). Targeting cPLA2 derived lipid hydroperoxides as a potential intervention for sarcopenia. Sci. Rep..

[2] Miyazawa T. (2021). Lipid hydroperoxides in nutrition, health, and diseases. Proc. Jpn. Acad. Ser. B.

[3] Zhong S., Li L., Shen X., Li Q., Xu W., Wang X., Tao Y., Yin H. (2019). An update on lipid oxidation and inflammation in cardiovascular diseases. Free Radic. Biol. Med..

[4] Nagashima T., Oikawa S., Hirayama Y., Tokita Y., Sekikawa A., Ishigaki Y., Yamada R., Miyazawa T. (2002). Increase of serum phosphatidylcholine hydroperoxide dependent on glycemic control in type 2 diabetic patients. Diabetes Res. Clin. Pract..

[5] Yoshida Y., Niki E. (2006). Bio-markers of lipid peroxidation in vivo: Hydroxyoctadecadienoic acid and hydroxycholesterol. Biofactors.

[6] Hong M.Y., Chapkin R.S., Barhoumi R., Burghardt R.C., Turner N.D., Henderson C.E., Sanders L.M., Fan Y.Y., Davidson L.A., Murphy M.E. (2002). Fish oil increases mitochondrial phospholipid unsaturation, upregulating reactive oxygen species and apoptosis in rat colonocytes. Carcinogenesis.

[7] Abeyrathne E.D.N.S., Nam K., Ahn D.U. (2021). Analytical Methods for Lipid Oxidation and Antioxidant Capacity in Food Systems. Antioxidants.

[8] Huang X., Ahn D.U. (2019). Lipid oxidation and its implications to meat quality and human health. Food Sci. Biotechnol..

[9] Papuc C., Goran G.V., Predescu C.N., Nicorescu V. (2017). Mechanisms of Oxidative Processes in Meat and Toxicity Induced by Postprandial Degradation Products: A Review. Compr. Rev. Food Sci. Food Saf..

[10] Hui S.P., Murai T., Yoshimura T., Chiba H., Nagasaka H., Kurosawa T. (2005). Improved HPLC assay for lipid peroxides in human plasma using the internal standard of hydroperoxide. Lipids.

[11] Hui S.P., Sakurai T., Ohkawa F., Furumaki H., Jin S., Fuda H., Takeda S., Kurosawa T., Chiba H. (2012). Detection and characterization of cholesteryl ester hydroperoxides in oxidized LDL and oxidized HDL by use of an Orbitrap mass spectrometer. Anal. Bioanal. Chem..

[12] HPLC analysis of lipid-derived polyunsaturated fatty acid peroxidation products in oxidatively modified human plasma. PubMed. https://pubmed.ncbi.nlm.nih.gov/10839772/.

[13] Misak A., Brezova V., Grman M., Tomasova L., Chovanec M., Ondrias K. (2020). •BMPO-OOH Spin-Adduct as a Model for Study of Decomposition of Organic Hydroperoxides and the Effects of Sulfide/Selenite Derivatives. An EPR Spin-Trapping Approach. Antioxidants.

[14] Merkx D.W.H., Swager A., van Velzen E.J.J., van Duynhoven J.P.M., Hennebelle M. (2021). Quantitative and Predictive Modelling of Lipid Oxidation in Mayonnaise. Antioxidants.

[15] Fereidoon S., Ying Z. (2010). Lipid oxidation and improving the oxidative stability. Chem. Soc. Rev..

[16] Ibusuki D., Nakagawa K., Asai A., Oikawa S., Masuda Y., Suzuki T., Miyazawa T. (2008). Preparation of pure lipid hydroperoxides. J. Lipid Res..

[17] Khoury S., Pouyet C., Lyan B., Pujos-Guillot E. (2018). Evaluation of oxidized phospholipids analysis by LC-MS/MS. Anal. Bioanal. Chem..

[18] MacMillan D.K., Murphy R.C. (1995). Analysis of lipid hydroperoxides and long-chain conjugated keto acids by negative ion electrospray mass spectrometry. J. Am. Soc. Mass Spectrom..

[19] Ahern K.W., Serbulea V., Wingrove C.L., Palas Z.T., Leitinger N., Harris T.E. (2019). Regioisomer-independent quantification of fatty acid oxidation products by HPLC-ESI-MS/MS analysis of sodium adducts. Sci. Rep..

[20] Hui S.P., Yoshimura T., Murai T., Chiba H., Kurosawa T. (2000). Determination of Regioisomeric Hydroperoxides of Fatty Acid Cholesterol Esters Produced by Photosensitized Peroxidation Using HPLC. Anal. Sci..

[21] Gowda S.G.B., Gao Z.J., Chen Z., Abe T., Hori S., Fukiya S., Ishizuka S., Yokota A., Chiba H., Hui S.P. (2020). Untargeted lipidomic analysis of plasma from high-fat diet-induced obese rats using UHPLC-Linear trap quadrupole-orbitrap MS. Anal. Sci..

[22] Siddabasave S.G., Ikeda K., Arita M. (2018). Facile determination of sphingolipids under alkali condition using metal-free column by LC-MS/MS. Anal. Bioanal. Chem..

[23] Ikuta A., Sakurai T., Nishimukai M., Takahashi Y., Nagasaka A., Hui S.P., Hara H., Chiba H. (2019). Composition of plasmalogens in serum lipoproteins from patients with non-alcoholic steatohepatitis and their susceptibility to oxidation. Clin. Chim. Acta.

[24] Gowda S.G.B., Gowda D., Ohno M., Liang C., Chiba H., Hui S.-P. (2021). Detection and Structural Characterization of SFAHFA Homologous Series in Mouse Colon Contents by LTQ-Orbitrap-MS and Their Implication in Influenza Virus Infection. J. Am. Soc. Mass Spectrom..

[25] Nakamura T., Maeda H. (1991). A simple assay for lipid hydroperoxides based on triphenylphosphine oxidation and high-performance liquid chromatography. Lipids.

[26] Chotimarkorn C., Ohshima T., Ushio H. (2006). Fluorescent image analysis of lipid hydroperoxides in fish muscle with 3-perylene diphenylphosphine. Lipids.

[27] Hicks M., Gebicki J.M. (1979). A spectrophotometric method for the determination of lipid hydroperoxides. Anal. Biochem..

[28] Tokumaru S., Tsukamoto I., Iguchi H., Kojo S. (1995). Specific and sensitive determination of lipid hydroperoxides with chemical derivatization into 1-naphthyldiphenylphosphine oxide and high-performance liquid chromatography. Anal. Chim. Acta.

[29] Turnipseed S.B., Allentoff A.J., Thompson J.A. (1993). Analysis of Trimethylsilylperoxy Derivatives of Thermally Labile Hydroperoxides by Gas Chromatography-Mass Spectrometry. Anal. Biochem..

[30] Hui S.P., Taguchi Y., Takeda S., Ohkawa F., Sakurai T., Yamaki S., Jin S., Fuda H., Kurosawa T., Chiba H. (2012). Quantitative determination of phosphatidylcholine hydroperoxides during copper oxidation of LDL and HDL by liquid chromatography/mass spectrometry. Anal. Bioanal. Chem..

[31] Kato S., Shimizu N., Hanzawa Y., Otoki Y., Ito J., Kimura F., Takekoshi S., Sakaino M., Sano T., Eitsuka T. (2018). Determination of triacylglycerol oxidation mechanisms in canola oil using liquid chromatography–tandem mass spectrometry. NPJ Sci. Food.

[32] Dussault P., Porter N.A. (1988). The resolution of racemic hydroperoxides: The preparation of optically pure hydroperoxide natural products. J. Am. Chem. Soc..

[33] Gardner H.W., Simpson T.D., Hamberg M. (1996). Mechanism of linoleic acid hydroperoxide reaction with alkali. Lipids.

[34] Bowry V.W., Stanley K.K., Stocker R. (1992). High density lipoprotein is the major carrier of lipid hydroperoxides in human blood plasma from fasting donors. Proc. Natl. Acad. Sci. USA.

[35] Kato S., Osuka Y., Khalifa S., Obama T., Itabe H., Nakagawa K. (2021). Investigation of lipoproteins oxidation mechanisms by the analysis of lipid hydroperoxide isomers. Antioxidants.

