# Supplementary material for: Simple and Sensitive Method for the Quantitative Determination of Lipid Hydroperoxides by Liquid Chromatography/Mass Spectrometry
Source: Antioxidants (Basel). 2022 Jan 25;11(2):229. doi: 10.3390/antiox11020229 (PMC8868426; doi:10.3390/antiox11020229)

Supporting information for

Simple and sensitive method for the quantitative determination of lipid hydroperoxides by liquid chromatography/mass spectrometry

Chongsheng Liang[1#], Siddabasave Gowda B. Gowda[2#], Divyavani Gowda[2], Toshihiro Sakurai[2], Iku Sazaki[1], Hitoshi Chiba[3], Shu-Ping Hui[1*]

1. Graduate School of Health Sciences, Hokkaido University, Kita-12, Nishi-5, Kita-Ku, Sapporo 060-0812, Japan
2. Faculty of Health Sciences, Hokkaido University, Kita-12, Nishi-5, Kita-Ku, Sapporo 060-0812, Japan
3. Department of Nutrition, Sapporo University of Health Sciences, Nakanuma, Nishi-4-3-1-15, Higashi-ku, Sapporo 007-0894, Japan.

*Email: keino@hs.hokudai.ac.jp

Synthesis of the FAOOH standards

a. Synthesis FA 18:1-OOH

About 100 mg of oleic acid was dissolved in pyridine with hematoporphyrin (0.005 eq) and irradiated with 200W tungsten lamp under bubbling O_2 gas for 75 min at 15℃. The reaction mixture was poured into ice water, acidified with 2M HCl and extracted twice with dichloromethane (DCM). The DCM layer was collected and washed with brine and dried over by Na_2SO_4. After evaporation, the residue was subjected to purification by silica gel column chromatography (hexane/ethyl acetate (v/v) = 48/2 to 35/15). The purified weight is about 4.8 mg of FA18:1-OOH. HR-ESI-MS calculated for $C_{18}H_{33}O_4$ $[M-H]^-$ is 313.2384, found, 313.2384 (0.00 ppm). R_f =0.65. ^{1}H-NMR (400 MHz, CDCl3) δ 5.81-5.72 (m, 1H), 5.41-5.33 (m, 1H), 4.30-4.24 (q, 1H, J= 6.4 Hz, 14.6 Hz), 2.37-2.33 (m, 2H), 2.11-2.06 (m, 2H), 1.66-1.60 (m, 3H), 1.46-1.27 (brs, 19H), 0.90-0.86 (m, 3H).

b. Synthesis FA 18:2-OOH

The synthesis was carried out by the similar protocol as described for FA 18:1-OOH. By using 100 mg linoleic acid as starting material about 19. 9 mg of FA18:2-OOH was obtained. HR-ESI-MS calculated for $C_{18}H_{31}O_4$ $[M-H]^-$ is 311.2228, found, 311.2228 (0.00ppm). R_f =0.65. ^{1}H-NMR (400 MHz, CDCl3) δ 6.60-6.53 (m, 1H), 6.03-5.98 (t, 1H), 5.60-5.48 (m, 2H), 4.41-4.35 (q, 1H, J= 6.1 Hz, 14.6 Hz), 4.15-4.10 (q,1H, J= 6.8 Hz, 14.2 Hz), 2.37-2.33 (m, 2H), 2.22-2.16 (m, 2H), 1.68-1.61 (m, 3H), 1.52-1.24 (brs, 16H), 0.90-0.86 (m, 3H).

c. Synthesis FA 18:3-OOH

The synthesis was carried out by the similar protocol as described for FA 18:1-OOH. By using 100 mg γ-linoleic acid as starting material about 14.9 mg FA18:3-OOH. was obtained. HR-ESI-MS calculated for $C_{18}H_{29}O_4$ [M-H]- is 309.2071, found, 309.2071 (0.00ppm). Rf =0.65.

d. Synthesis FA 19:1-OOH

The synthesis was carried out by the similar protocol as described for FA 18:1-OOH. By using 100 mg nonadecenoic acid as starting material about 28.3 mg FA 19:1-OOH. was obtained. HR-ESI-MS calculated for $C_{19}H_{35}O_4$ $[M-H]^-$ is 327.2541, found, 327.2540 (-0.31ppm). R_f =0.75. ^{1}H-NMR (400 MHz, CDCl3) δ 5.81-5.74 (m, 1H), 5.40-5.34 (m, 1H), 4.30-4.24 (q, 1H, J= 6.4 Hz, 14.6 Hz), 2.37-2.33 (m, 2H), 2.11-2.05 (m, 2H), 1.65-1.60 (m, 3H), 1.46-1.27 (brs, 21H), 0.90-0.86 (m, 3H).

e. Synthesis FA 20:4-OOH

The synthesis was carried out by the similar protocol as described for FA 18:1-OOH. By using 76 mg arachidonic acid as starting material about 18:9 mg FA 20:4-OOH was obtained. HR-ESI-MS calculated for $C_{20}H_{31}O_4$ $[M-H]^-$ is 335.2228, found, 335.2228 (0.00ppm). R_f =0.63.

f. Synthesis FA 20:5-OOH

The synthesis was carried out by the similar protocol as described for FA 18:1-OOH. By using 100 mg eicosapentaenoic acid as starting material about 13.8 mg FA 20:5-OOH was obtained. HR-ESI-MS calculated for $C_{20}H_{29}O_4$ $[M-H]^-$,333.2071, found, 333.2071 (0.00ppm). R_f =0.63.

g. Synthesis FA22:1-OOH

The synthesis was carried out by the similar protocol as described for FA 18:1-OOH. By using 213 mg erucic acid as starting material about 15.2 mg FA 22:1-OOH was obtained. HR-ESI-MS calculated for $C_{22}H_{41}O_4$ $[M-H]^-$, 369.3010, found, 369.3008 (-0.54ppm). R_f =0.75. ^{1}H-NMR (400 MHz, CDCl3) δ 5.81-5.73 (m, 1H), 5.40-5.33 (m, 1H), 4.30-4.24 (q, 1H, J= 6.8 Hz, 14.6 Hz), 2.37-2.33 (m, 2H), 2.11-2.05 (m, 2H), 1.67-1.62 (m, 3H), 1.42-1.26 (brs, 27H), 0.90-0.86 (m, 3H).

h. Synthesis FA22:6-OOH

The synthesis was carried out by the similar protocol as described for FA 18:1-OOH. By using 37.5mg docosahexaenoic acid as starting material about 5.4mg FA22:6-OOH was obtained. HR-ESI-MS calculated for $C_{22}H_{31}O_4$ $[M-H]^-$,359.2228, found, 359.2228 (0.00ppm). R_f =0.65.

2. Synthesis of the FAOOMxP standards

a. Synthesis of FA18:1-OOMxP

A two-step reaction was conducted to prepare FA18:1-OOMxP. At first, the FA 18:1-OOH was prepared by the above-described photochemical oxidation using 500 mg of oleic acid. Then, the FA18:1-OOH was dissolved in dichloromethane and reacted with excess of 2-Methoxypropene (6 ml) in presence of pyridinium *p*-Toluene sulfonate (3 mg) for 10 min at room temperature. The reaction mixture was poured into Milli-Q and extracted twice with dichloromethane (DCM). The DCM layer was collected and washed with brine and dried over by Na_2SO_4. After evaporation, the residue was subjected to purification by silica gel column chromatography (hexane/ethyl acetate (v/v) = 48/2 to 35/15) to obtain 25.9 mg of FA18:1-OOMxP. HR-ESI-MS calculated for $C_{22}H_{42}NaO_5$ $[M+Na]^+$ is 409.2924, found, 409.2917 (-1.71ppm). R_f =0.75. ^{1}H-NMR

(400 MHz, CDCl3) δ 5.69-5.60 (m, 1H), 5.42-5.35 (m, 1H), 4.32-4.26 (q, 1H), 3.30 (s, 3H), 2.37-2.32 (m, 2H), 2.08-2.03 (m, 2H), 1.67-1.62 (m, 3H), 1.44-1.26 (brs, 24), 0.90-0.87 (m, 3H).

b. Synthesis of FA18:2-OOMxP

The synthesis was conducted similar to FA 18:1-OOMxP using 500 mg linoleic acid as a starting material to yield 29.7 mg of FA 18:2-OOMxP as product. HR-ESI-MS calculated for $C_{22}H_{40}NaO_5$ $[M+Na]^+$ is 407.2768, found, 407.2761 (-1.72ppm). R_f =0.75. ^{1}H-NMR (400MHz, CDCl3) δ 6.50-6.43(m, 1H), 6.01-5.95(m, 1H), 5.62-5.57 (m, 1H), 5.46-5.40 (m, 1H), 4.42-4.36 (q, 1H, J=6.3 Hz, 14.2 Hz), 3.29-3.25 (m, 3H), 2.35-2.31 (m, 2H), 2.19-2.14 (m, 2H), 1.68-1.60 (m, 3H), 1.47-1.28 (brs, 21), 0.88-0.85 (m, 3H).

c. Synthesis of FA18:3-OOMxP

The synthesis was conducted similar to FA 18:1-OOMxP using 100 mg γ-linoleic acid as a starting material to yield 5.5 mg of FA 18:3-OOMxP as product. HR-ESI-MS calculated for $C_{22}H_{38}NaO_5$ $[M+Na]^+$is 405.2611, found, 405.2603 (-1.97ppm). R_f =0.75.

d. Synthesis of FA19:1-OOMxP

The synthesis was conducted similar to FA 18:1-OOMxP using 150 mg nonadecenoic acid as a starting material to yield 19 mg of FA 19:1-OOMxP as product. HR-ESI-MS calculated for $C_{23}H_{44}NaO_5$ $[M+Na]^+$ is 423.3081, found, 423.3081 (0.00ppm). R_f =0.83. ^{1}H-NMR (400 MHz, CDCl3) δ 5.67-5.61 (m, 1H), 5.41-5.36 (m, 1H), 4.32-4.26 (q, 1H, J=6.4 Hz, 14.2 Hz), 3.30 (s, 3H), 2.36-2.33 (m, 2H), 2.08-2.03 (m, 2H), 1.67-1.62 (m, 3H), 1.39-1.26 (brs, 28H), 0.90-0.86 (m, 3H).

e. Synthesis of FA20:4-OOMxP.

The synthesis was conducted similar to FA 18:1-OOMxP using 100 mg arachidonic acid as a starting material to yield 14.3 mg of FA 20:4-OOMxP as product. HR-ESI-MS calculated for $C_{24}H_{40}NaO_5$ $[M+Na]^+$ is 431.2768, found, 431.2761 (-1.62ppm). R_f =0.80.

f. Synthesis of FA20:5-OOMxP

The synthesis was conducted similar to FA 18:1-OOMxP using 100 mg eicosapentaenoic acid as a starting material to yield 13.6 mg of FA 20:5-OOMxP as product. HR-ESI-MS calculated for $C_{24}H_{38}NaO_5$ $[M+Na]^+$ is 429.2611, found, 429.2603. (-1.86ppm). R_f=0.78.

g. Synthesis of FA22:1-OOMxP

The synthesis was conducted similar to FA 18:1-OOMxP using 500 mg erucic acid as a starting material to yield 43.4 mg of FA 22:1-OOMxP as product. HR-ESI-MS calculated for $C_{26}H_{50}NaO_5$ $[M+Na]^+$ is 465.3550, found, 465.3549 (-0.21ppm). R_f=0.85. 1H-NMR (400 MHz, CDCl3) δ 5.68-5.61 (m, 1H), 5.41-5.35 (m, 1H), 4.32-4.26 (q, 1H, J=6.4 Hz, 13.7 Hz), 3.30 (s, 3H), 2.36-2.32 (t, 2H, J=7.3 Hz, 15.1 Hz), 2.08-2.03 (q, 2H J=7.3 Hz, 14.6 Hz), 1.67-1.59 (m, 3H), 1.44-1.27 (brs, 33H), 0.90-0.86 (m, 3H).

h. Synthesis of FA22:6-OOMxP

The synthesis was conducted similar to FA 18:1-OOMxP using 100 mg docosahexaenoic acid as a starting material to yield 21.3 mg of FA 22:6-OOMxP as product. The HR-ESI-MS calculated for $C_{26}H_{40}NaO_5$ $[M+Na]^+$ is 455.2768, found, 455.2760 (-1.76ppm). R_f =0.78.

3. Mass and ¹H NMR spectra of FAOOH and its 2-MxP derivatives

a. FA 18:1-OOH

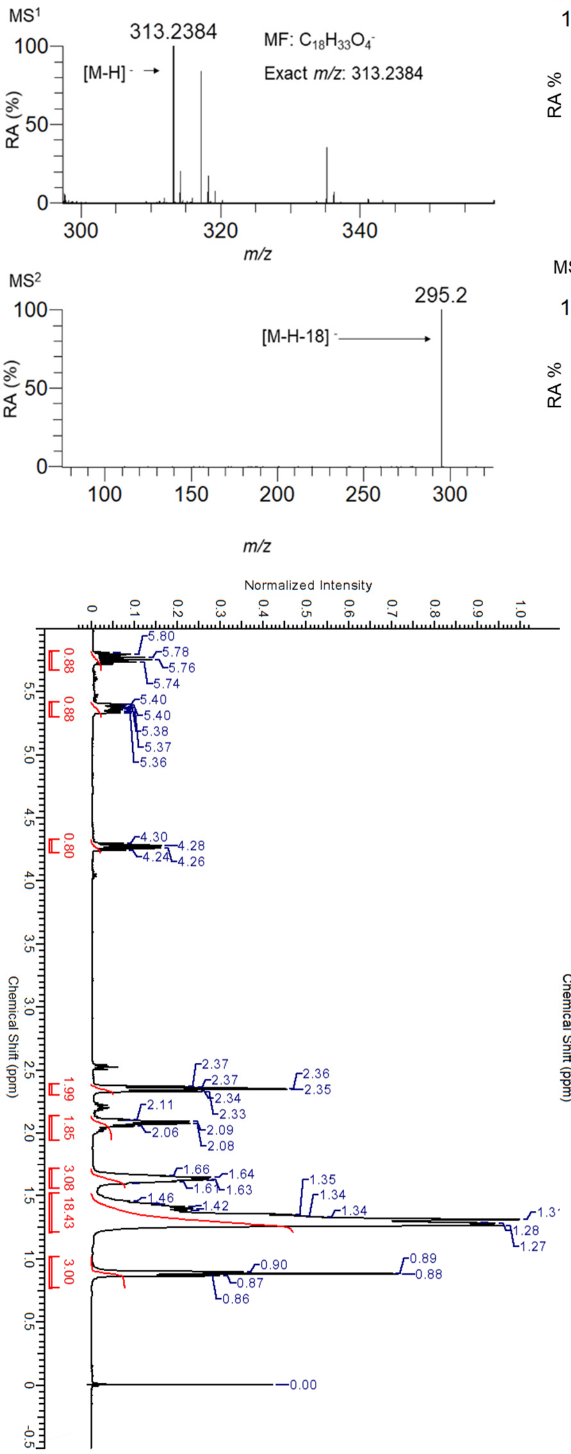

b. FA 18:1-OOMxP

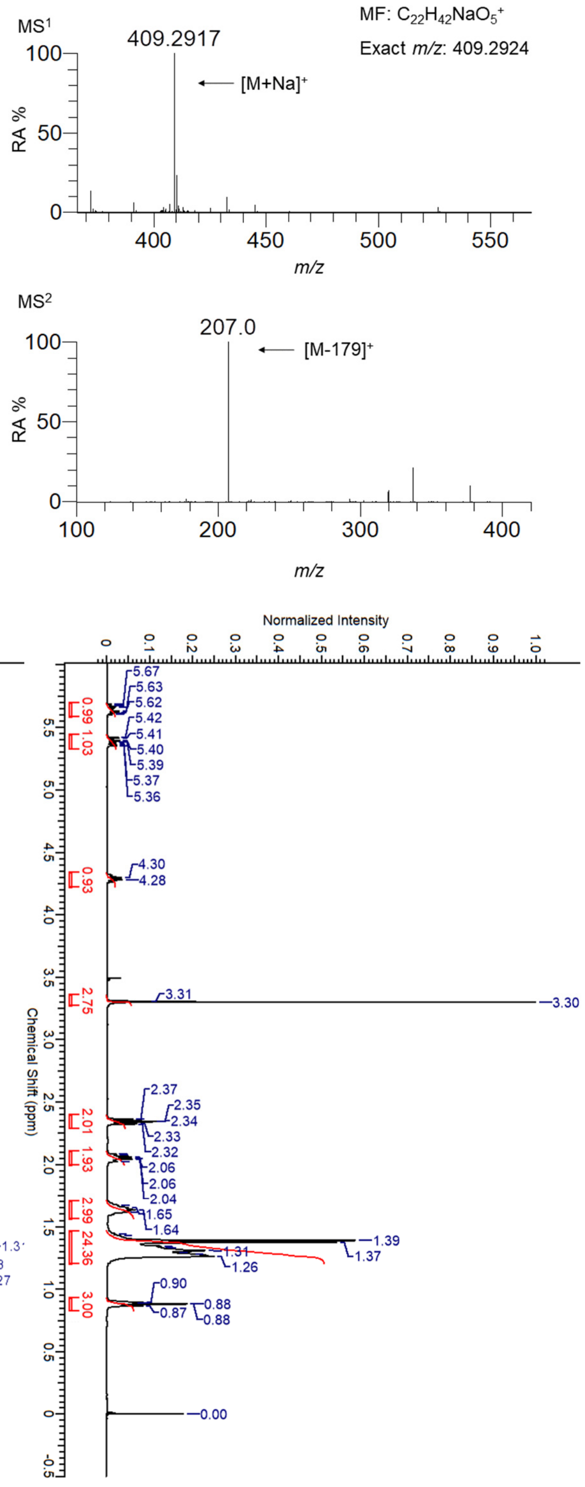

c. FA 18:2-OOH

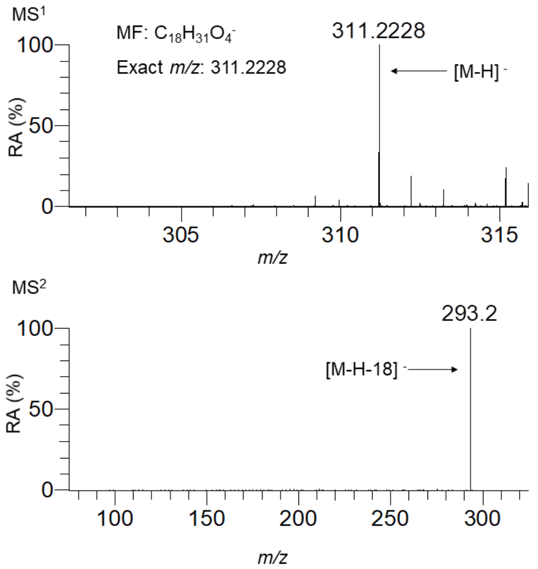

d. FA 18:2-OOMxP

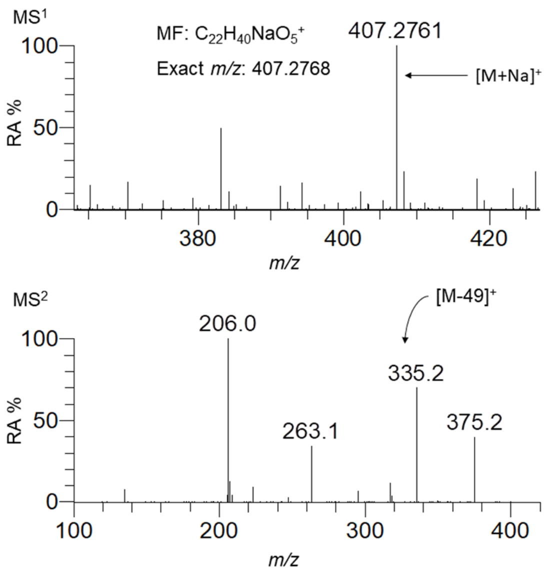

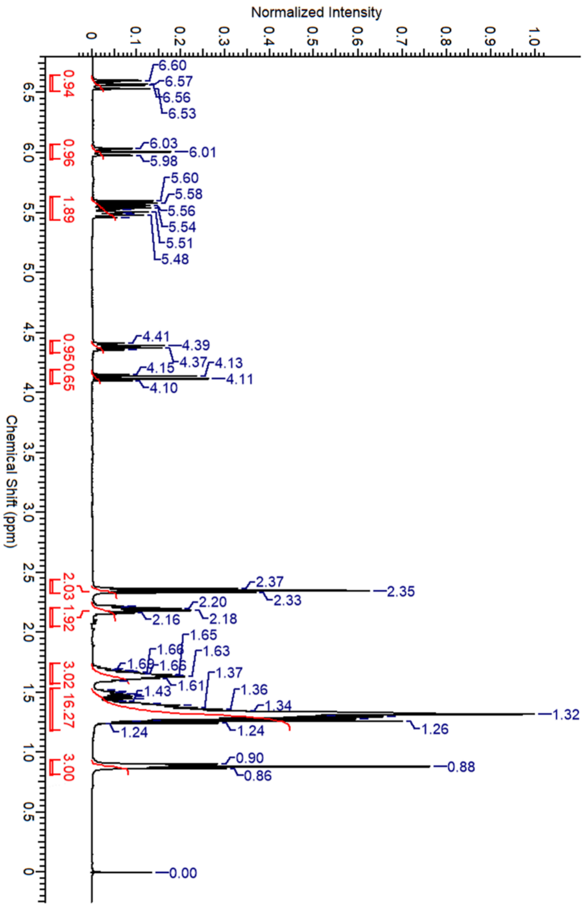

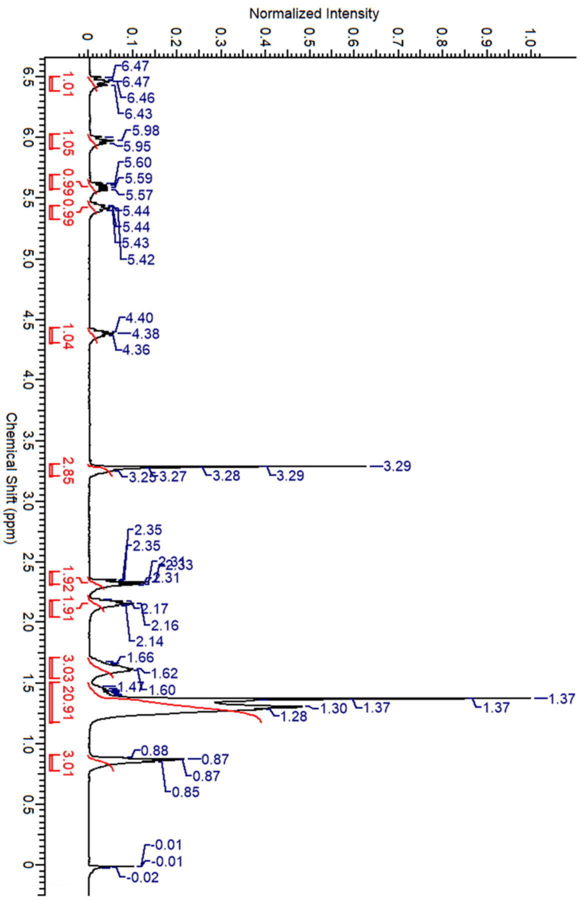

e. FA 19:1-OOH

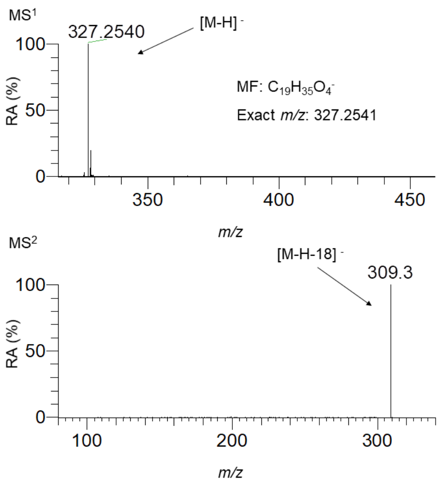

f. FA 19:1-OOMxP

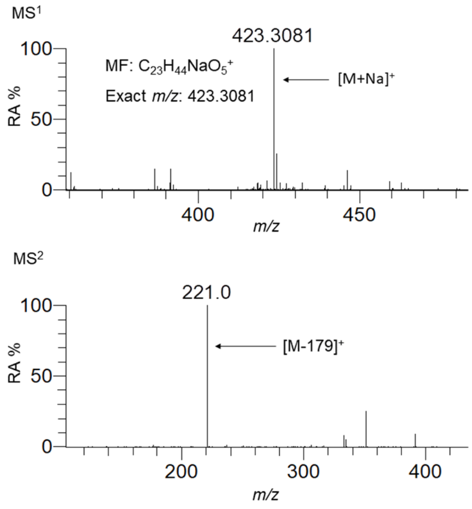

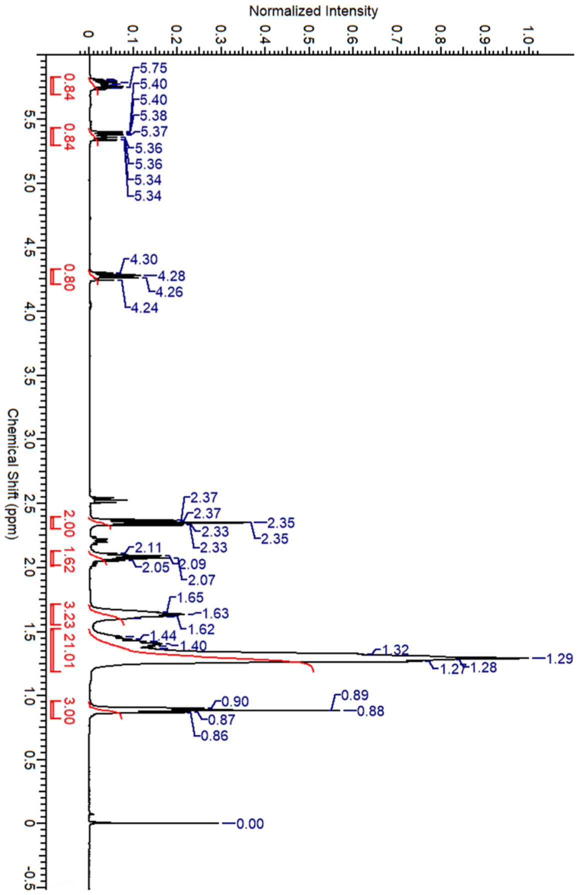

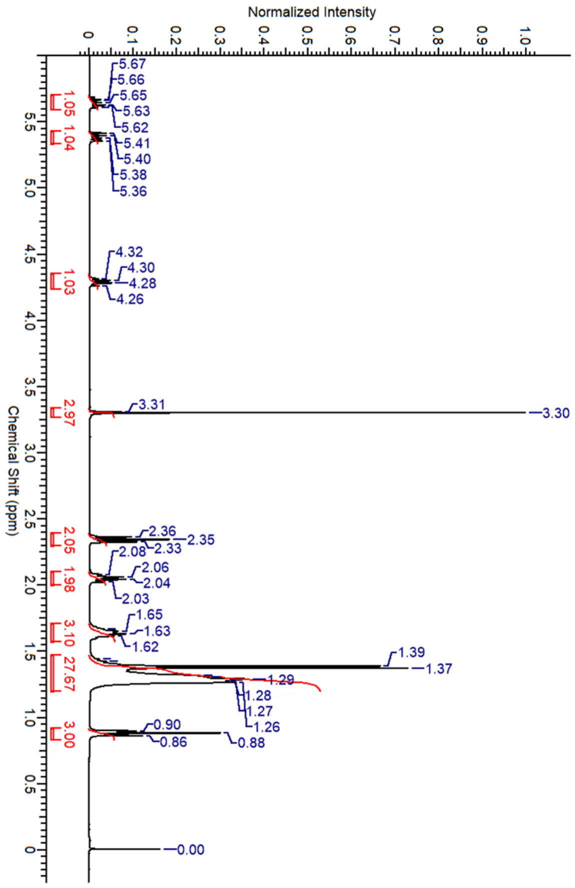

g. FA 22:1-OOH

h. FA 22:1-OOMxP

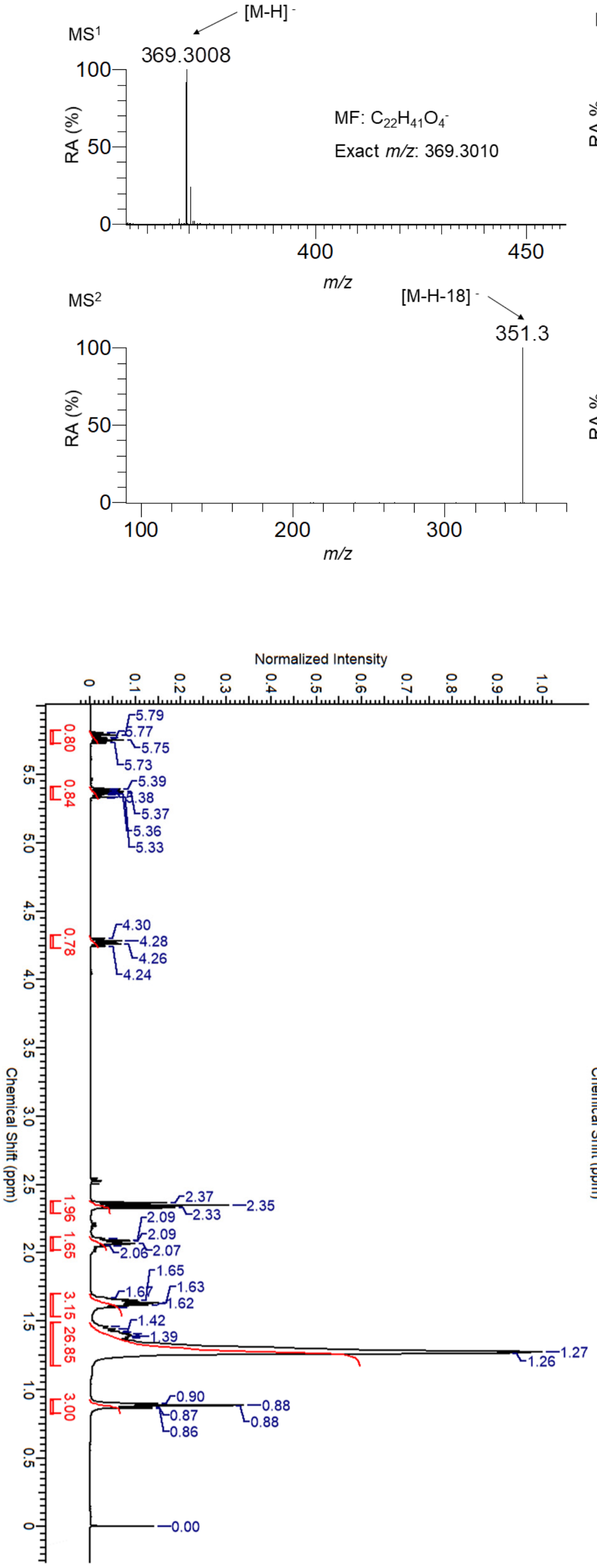

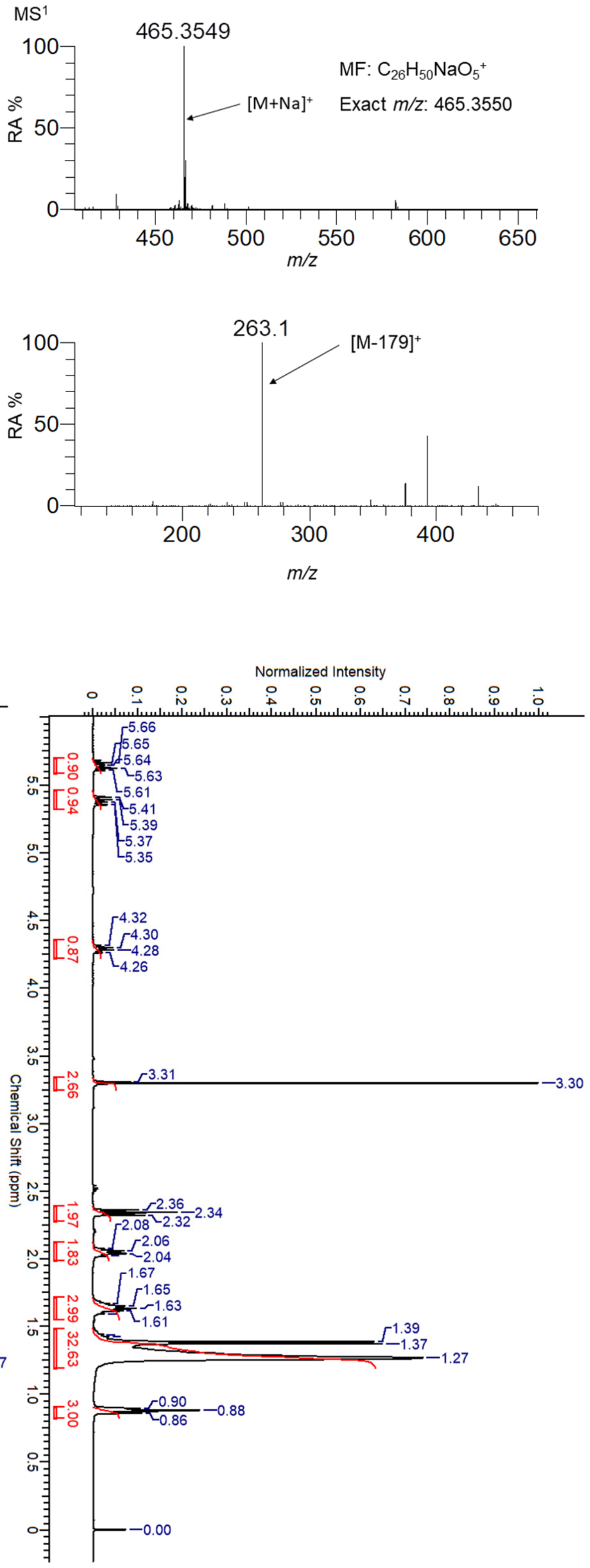

i. FA 18:3-OOH

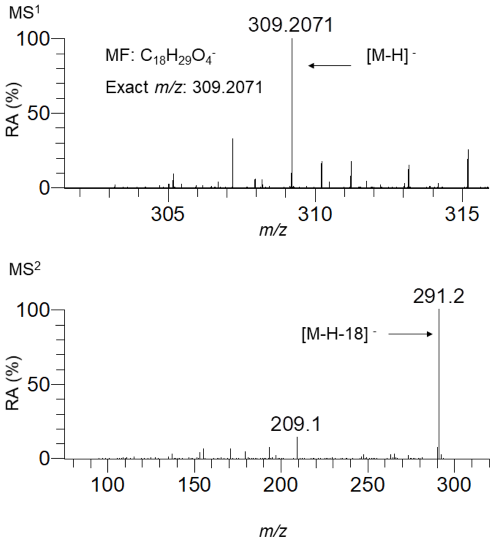

j. FA 18:3-OOMxP

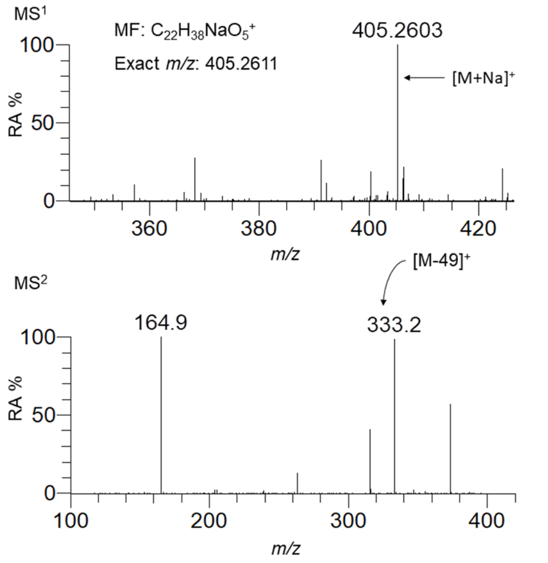

k. FA 20:4-OOH

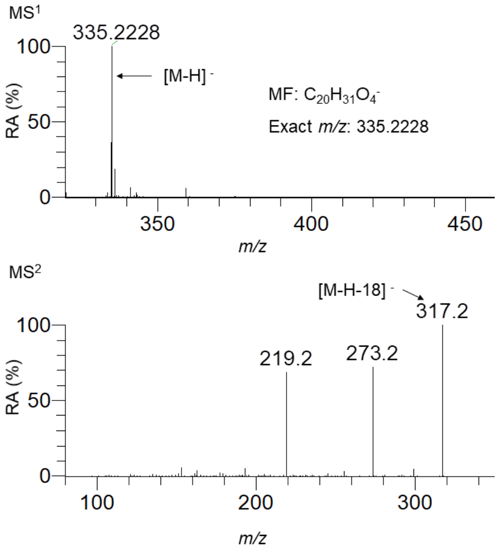

l. FA 20:4-OOMxP

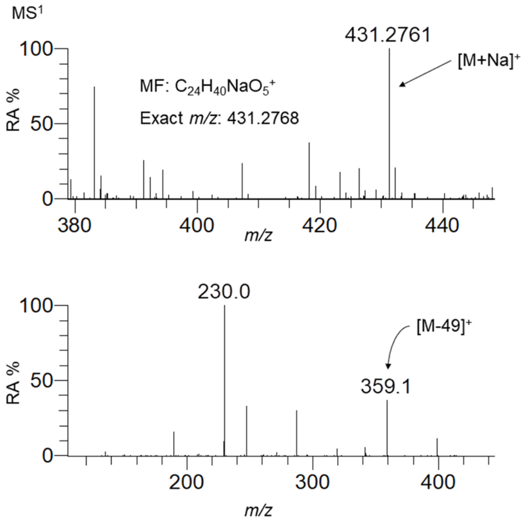

m. FA 20:5-OOH

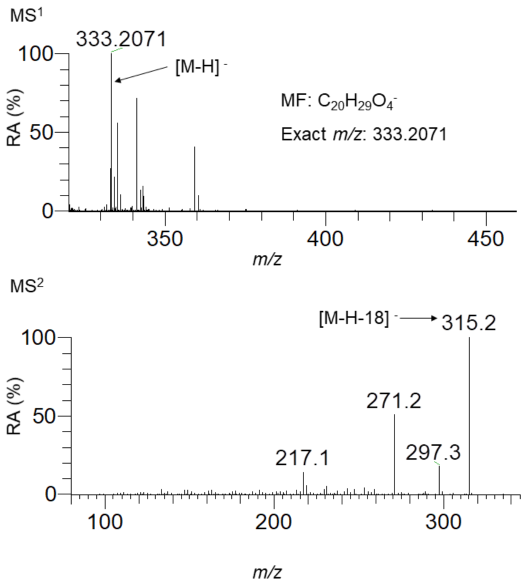

n. FA 20:5-OOMxP

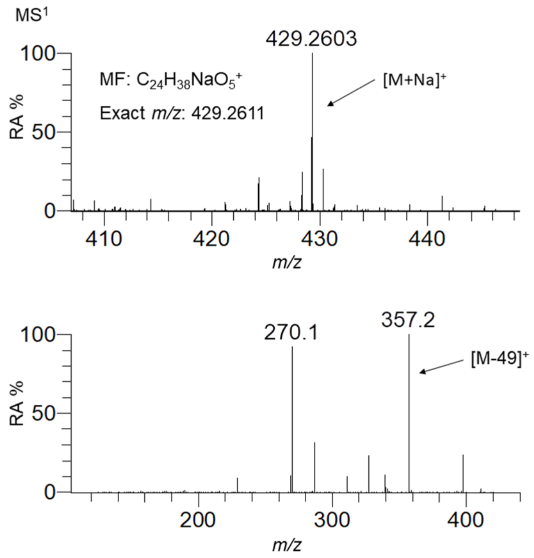

o. FA 22:6-OOH

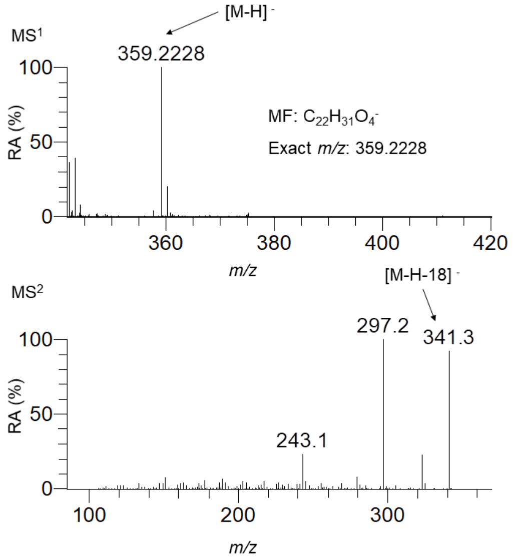

p. FA 22:6-OOMxP

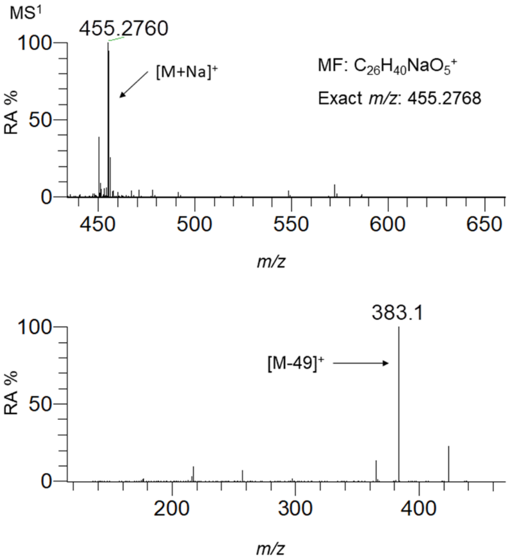

4. Optimization of reagents for FAOOHs MxP derivatization.

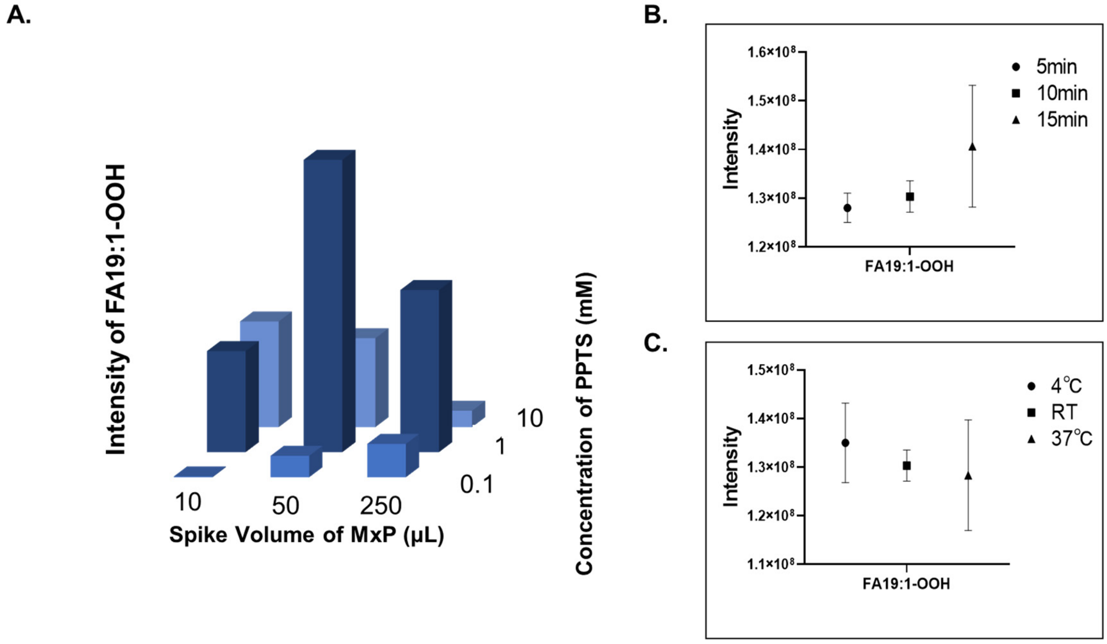

A. Changes in intensity level with variation of volume of 2-MxP and amount of PPTS added. **B**. Changes is intensities with respect to time **C**. Changes is intensities with respect to temperature with plasma matrix. (n=3).

5. The stability of FAOOMxPs in 1M KOH with plasma matrix

Species	Control (n=4)		After hydrolysis (n=4)		After hydrolysis/control (%)
	Average	RSD (%)	Average	RSD (%)	
18:1-OOMxP	8.49E+07	4.2	8.22E+07	8.1	96.9
18:2-OOMxP	1.59E+07	1.1	1.65E+07	2.1	103.5
18:3-OOMxP	2.36E+07	4.8	2.17E+07	4.7	92.0
20:4-OOMxP	8.63E+06	5.2	9.77E+06	6.9	113.1
20:5-OOMxP	1.87E+07	4.4	1.81E+07	2.8	96.9
22:1-OOMxP	5.19E+07	1.5	5.46E+07	1.8	105.2
22:6-OOMxP	1.02E+07	3.5	1.17E+07	5.2	114.7
19:1-OOMxP	4.88E+07	11.4	5.43E+07	9.0	111.4

6. Chromatograms of FAOOMxPs before and after derivatization in biological samples

A.

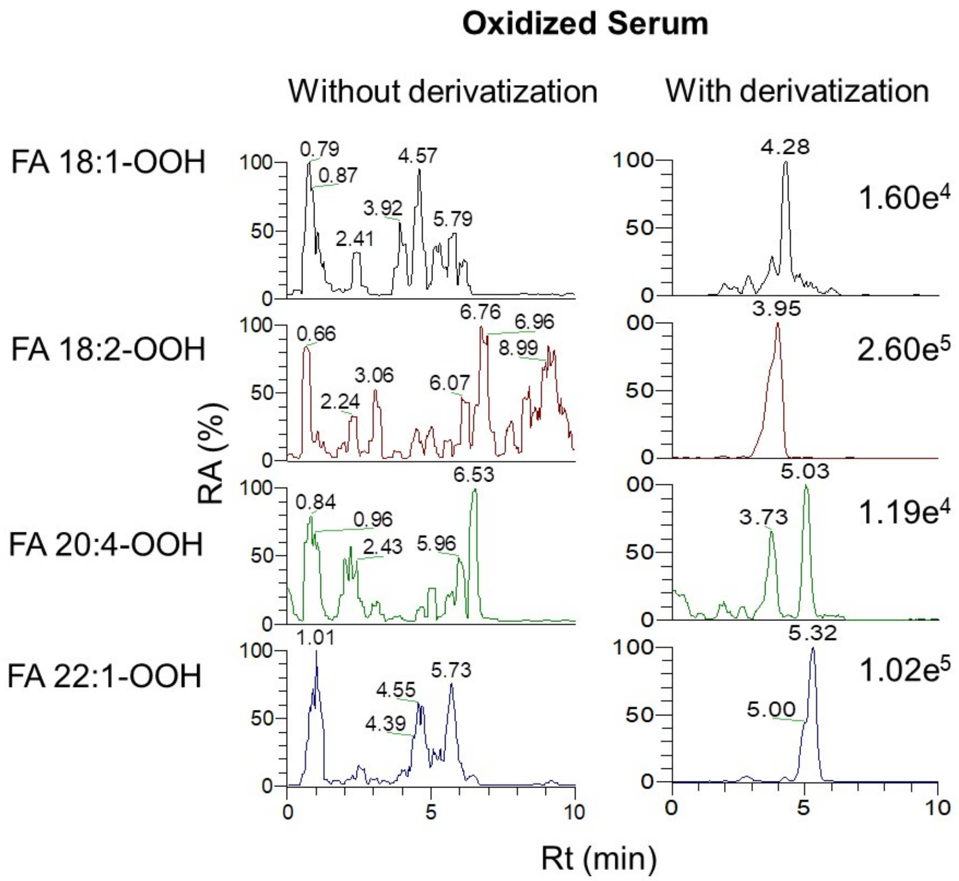

B.

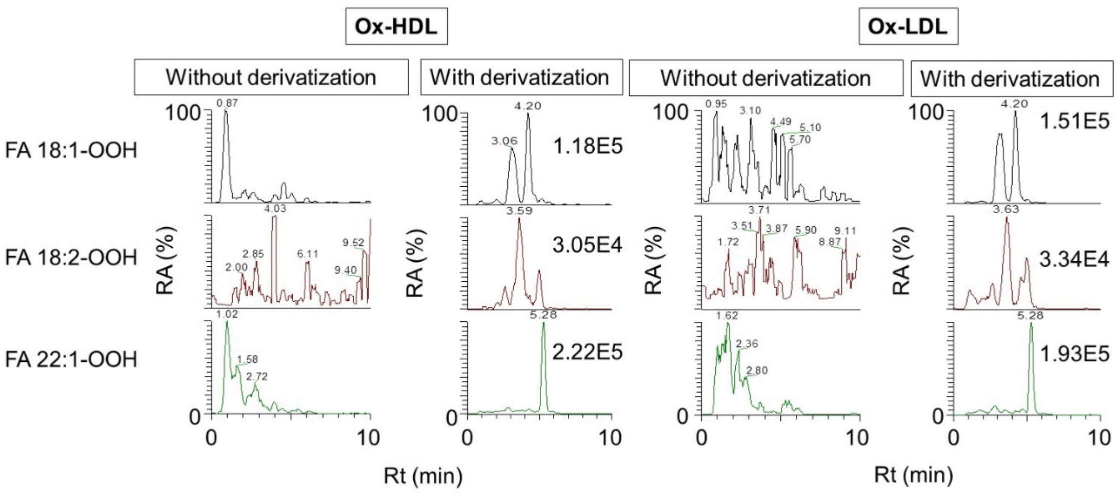

Supplement: Supplementary file 1 [file antioxidants-11-00229-s001.zip › antioxidants-1507760-supplementary.pdf]
